# Shaping industrial spatial density: How floor area ratio varies across regions and sectors in Zhejiang, China

**DOI:** 10.1371/journal.pone.0343089

**Published:** 2026-03-04

**Authors:** Fan Tu, Ruijia Ji, Jinyu Zhou, Gabriel Hoh Teck Ling

**Affiliations:** 1 Department of Urban and Rural Planning, School of Design and Architecture, Zhejiang University of Technology, Hangzhou, China; 2 Housing and Urban-Rural Development Bureau of Jianshui County, Yunnan, China; 3 Department of Urban and Regional Planning, Faculty of Built Environment and Surveying, University Teknologi Malaysia, Malaysia; Zhejiang A and F University, CHINA

## Abstract

Spatial density plays a pivotal role in influencing land-use patterns and land consumption. To meet evolving industrial needs and maximize limited land resources, incorporating floor area ratio (FAR) optimization into urban planning is essential. Existing studies on floor area ratio largely focus on residential land, overlooking how industrial FAR varies across regions and sectors and what drives these differences. This study addresses this gap by analyzing newly supplied industrial land data from 90 counties in Zhejiang Province, China (2019–2023), using panel regression analysis. The findings reveal a steady FAR increase of 6.8% annually, with the highest levels in the northern coastal cities Hangzhou and Ningbo, a mid-range peak around Jinhua, and relatively lower but rising values toward the southern areas including Wenzhou. FAR is positively associated with land prices and government land supply, but negatively with per capita arable land. Significant regional and industrial disparities are observed, shaped by geography, land price and industry structure. These findings underscore the need for adaptive FAR policies that balance land efficiency and industrial compatibility. The study recommends stabilizing industrial land prices, supporting clustered small and micro enterprises with higher-density development, and selectively implementing the Industry’s Going Upstairs (IGU) strategy based on structural load, production characteristics, and suitability for mixed-use.

## Introduction

Spatial density is a core concept for measuring the intensity of urban spatial development and the capacity of functional carrying. It has long been regarded as a fundamental variable in studies of urban morphological evolution, land-use efficiency, and spatial planning. Among relevant indicators, Floor Area Ratio (FAR)—the ratio of total building floor area to the land area it occupies—not only represents the three-dimensional development intensity of urban form, but also reflects the carrying capacity of land, making it a key indicator for assessing spatial density. When FAR fails to align with market demand or match spatial resource endowments, it can result in a mismatch between development intensity and land value, leading to imbalances of "high" or "low" spatial density [1]. For example, urban sprawl in Bangalore, India is closely associated with persistently low FAR settings [2], while the low population density in central Moscow reflects insufficient FAR allocation under non-market mechanisms [3]. FAR has great impact on urban density, space structure, housing price, firm growth, pollution, disaster risks, urban infrastructure and social segregation [2,3–7].

Alonso’s bid-rent theory [8] laid the foundation for understanding the spatial distribution of economic activities. Building on this framework, the floor area ratio (FAR) has been interpreted as a proxy for the capital–land ratio, reflecting developers’ optimization of building form in response to relative input prices [9]. Most existing studies, however, have concentrated on residential land and urban contexts [10–12]. Within the limited research on industrial spatial density, Wei and Zhang [13] provide valuable insights through their case study of Shenzhen, revealing how the "Industrial Growing Upstairs (IGU)" initiative was shaped by concerns over manufacturing out-migration and competitive pressures amid volatile U.S.–China relations. Behrens et al. [14] further demonstrate that manufacturing establishments tend to use land more intensively in larger cities and central areas, and that larger firms generally exhibit higher employment-to-parcel-size ratios. Since existing evidence is often limited to single-city cases, offering fragmented insights into how industrial land use intensity evolves under changing economic and policy environments. Therefore, the dynamics and determinants of industrial floor area ratio (FAR) remain underexplored in the international literature. This study addresses this empirical and conceptual gap by developing a region-scale analytical framework based on 10,601 industrial land transactions across 90 counties in Zhejiang Province, China (2019–2023).

Zhejiang, ranking fourth in China by industrial added value, represents a globally relevant example of an advanced manufacturing region driven by small and micro enterprises—56,828 industrial firms in 2023, 84% of which are privately owned [15].The province's ongoing transformation toward innovation-intensive and advanced manufacturing, exemplified by firms such as Unitree Robotics and DeepSeek in Hangzhou, makes it an ideal case for examining how industrial FAR responds to diverse natural conditions, economic dynamics, and land policy regimes.

Accordingly, this study is guided by three research questions:(1) How has industrial FAR evolved over time in rapidly industrializing regions? (2) How does industrial FAR vary across city characteristics and industrial sectors? (3) What factors drive these spatial disparities?

This paper makes three key contributions to the literature on industrial spatial density. First, it offers an empirical analysis of industrial spatial density through the lens of FAR in rapidly industrializing regions. Second, it systematically identifies the determinants of industrial density and develops a framework to explain the interaction between industrial restructuring and spatial policy in contexts of industrial land intensification globally. Third, by identifying sectoral and locational differences in FAR, it clarifies varied spatial needs and offers comparative insights for industrial land planning globally.

The remainder of the paper is structured as follows. The literature review is illustrated in Section 2. Section 3 contextualizes China's industrial land-use intensification through historical policy trajectories. Section 4 delineates the methodological framework, including data sources and study area. The empirical results and analysis are presented in Section 5. The discussion and conclusion are presented in Section 6 and 7, respectively.

### Literature review

#### Urban Density: measures and the role of FAR.

Urban density is a key concept for describing the spatial structure and development intensity of cities. To capture its multidimensional nature, researchers have developed a range of indicators across economic, social and ecological dimensions. Economically, density is often measured by land productivity and spatial effectiveness—the value added or GDP per unit of land area [16]. On the social dimension, early work by Clark [17] introduced the negative exponential function to model population density patterns, while later studies, such as Small and Song [18]and Duranton and Puga [19]employed population and employment density as key indicators. In recent years, studies have moved beyond single-dimensional metrics to adopt composite measures such as land use efficiency, 'Vertical-to-Horizontal Growth' ratio,while some researches also integrate environmental performance [12,20–22].

Although economic, social, and ecological density have been widely studied, FAR—an essential indicator of land-use density—captures both regulatory limits and actual development intensity. FAR reflects vertical land use and influences outcomes such as productivity, spatial structure, and environmental performance. Governments in market economies use density regulations to shape urban form. For instance, in the United States, minimum lot size requirements are used to restrict suburban densities, while height limits control central city intensities [2]. FAR policies also play a central role in this regulatory toolkit, influencing the distribution of development between urban cores and peripheries. Simulation results by Bertaud and Brueckner [2] show that FAR limits can impose welfare losses equivalent to 2% of household income in urban areas. Bovet et al. [23]emphasized that FAR as a lever to promote compact, efficient development, thereby mitigating sprawl. In China, FAR thresholds have been used to constrain the oversupply of low-cost land and to address inefficient land use in sprawling urban peripheries [24]. Empirical studies also show that increased FAR is associated with reduced carbon emissions [25,26] and mitigates environmental injustice [27]. To emphasize this spatial dimension, this study uses "spatial density" to refer specifically to FAR, distinguishing it from density measures rooted in economic or social variables.

### Industrial spatial density: changes and regional variations

To accommodate evolving industrial needs and maximize limited land resources, new approaches to industrial land intensification have emerged. These include the development of industrial real estate in the form of steel-frame structures, commercial-industrial complexes, and multilevel buildings, significantly increasing floor area within the same land footprint [28]. Due to the scarcity of land resources, the demand for industrial upgrading, and urban industrialization, multi-story industrial buildings have emerged in places like Singapore and China [13,6,29] The push for higher density is largely driven by advancements in technology and the rapid expansion of e-commerce, which demand more complex spatial configurations. For instance, distribution centers now feature high ceilings for vertical racking systems, while automation technologies and multi-shift operations require more compact, yet functionally diverse, building layouts. Additionally, firms are increasingly integrating manufacturing, design, display, and logistics functions within a single high-density facility, further reinforcing the trend toward intensified industrial land use.

Local governments are crucial in driving the intensification of industrial land use through spatial planning, zoning policies, and regulations aimed at boosting land efficiency and productivity [28]. For instance, Vancouver Metro 2040 requires municipalities to implement strategies that promote better utilization and intensification of industrial zones. In London, the focus has shifted to high-density, mixed-use developments, particularly vertical industrial stacking, with the city recently creating a matrix to determine suitable uses for vertical intensification. The Port of Rotterdam in the Netherlands emphasizes land clustering and optimization for industrial intensification. Similarly, several North American cities are re-evaluating their older industrial buildings, converting them into modern industrial spaces rather than non-industrial uses. As economies evolve, land use and building requirements adapt accordingly. Hong Kong serves as an example, where industrial activities have shifted to mainland China, leading to the repurposing of former industrial buildings into commercial and office spaces [30].

In China, the shift towards vertical development represents a transformative shift in industrial land-use strategies, driven by the Industrial Growing Upstairs（IGU）model. Vertical expansion has emerged as a solution to land scarcity, optimizing spatial efficiency while supporting innovation in high-value-added industries. Shenzhen's IGU strategy has been instrumental in mitigating land constraints and fostering manufacturing sector resilience [8]. Studies across 320 Chinese cities demonstrate that vertical development significantly increases built-up area density and optimizes land use [31].

There is a large literature on FAR of housing and urban overall, but little known work that directly addresses the industrial FAR. A critical gap remains in understanding the factors that influence FAR of industrial land use. Some studies have contributed indirectly by investigating industrial land values, providing insights into how factors such as neighborhood attributes, locational advantages, and industrial agglomeration affect land use outcomes [32,33]. Benjamin et al. categorized prior research into several key dimensions: property characteristics, demand determinants, rent and income factors, valuation and return issues, environmental considerations, international comparisons, and management and financing concerns [34]. Tu et al. found that industrial land prices are 22% higher in national-level and 15% higher in provincial-level development zones than outside them, that higher building density raises prices, and that parcels in central urban areas command about 10% premiums over peripheral locations [33].

This study extends the limited theoretical understanding of industrial land use intensity by examining the determinants of FAR. It introduces an integrated perspective that links price dynamics, land supply mechanisms, and geographical conditions, thereby broadening the conceptual framework for analyzing how industrial density evolves under diverse regional and policy contexts.

### Theoretical framework

Current approaches to measuring industrial land use primarily emphasize economic output, using the concept of industrial land use efficiency. This economic-driven evaluation framework arises from land's intrinsic role as a capitalized asset [35]. However, evaluations that rely solely on economic output fail to capture how land is organized spatially, highlighting the need for an integrated spatial morphology perspective. The spatial density is reflected in the extent of intensive utilization of three-dimensional spatial resources, with the FAR serving as a key quantitative indicator that directly reflects the spatial development intensity of land. The spatial heterogeneity of FAR helps understand the factors influencing industrial land-use intensity.

To analyze the factors influencing FAR, it is essential to consider land price, government supply mechanisms, and topographical conditions as a critical analytical framework. Land price directly shapes industrial production costs. Government regulation of land supply determines land availability. Topography imposes physical constraints on feasible development intensity. Based on this analytical framework, the following sections elaborate on how price, regulatory supply mechanisms, and terrain conditions individually and jointly affect FAR.

Land price, as a critical determinant of production costs, directly influences the investment, upgrading, and relocation decisions of industrial enterprises. The marketisation of industrial land and fluctuations in its price not only alter the land supply structure but also directly affect land intensity. Lin et al. [36]demonstrated that the price mechanism induced by land marketization regulates resource allocation: higher industrial land prices, functioning as market signals, improved land use intensity, output per unit, and total factor productivity. Zhang et al. [37]conducted a micro-level enterprise analysis showing that industrial land prices affect enterprise demand for industrial land. These indicate that, as a production factor, land's price reflects supply-demand dynamics and serves as a crucial tool for governmental regulation of economic efficiency.

The government, as the owner and regulator of urban land resources, strictly controls land supply through the land use quota system in China. Local governments develop annual land supply plans based on national economic and social development goals, spatial planning, and overall land utilization strategies, while taking into account market demand and the availability of land resources [33]. Confronted with increasingly limited land resources, both the government and enterprises tend to promote more intensive land use by increasing the FAR [13]. Following the 2006 Regulation on "Transferring the Use Right of State-Owned Land by Tender, Auction, and Listing, land transfers must follow standardized public bidding, auction, or listing procedures". Policies explicitly set minimum FAR requirements for industrial land, thereby encouraging enterprises to develop in three-dimensional space, while increasing FAR during development does not require additional land transfer fees. Regions with limited land availability tend to adopt higher FAR policies to optimize spatial efficiency [38].

**Fig 1:**
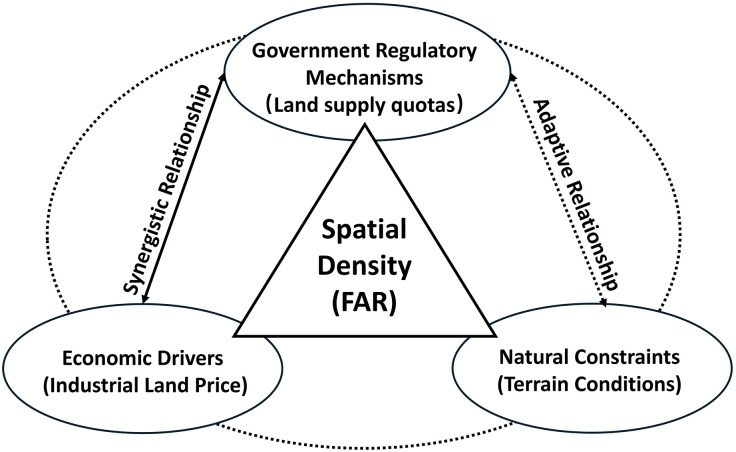
Key factors shaping industrial spatial density (FAR).

Terrain conditions play a crucial role in shaping land-use intensity. Areas with flat terrain generally allow for higher-density developments, as construction and land consolidation are relatively easier and less costly. In contrast, regions characterized by hills, slopes, or complex topography face constraints that limit the feasibility of dense, vertical development, resulting in lower FAR. Consequently, policymakers and enterprises need to incorporate terrain considerations into land allocation strategies, with the practical constraints imposed by natural geography (Fig 1).

### Historical background of China's industrial land intensification and vertical development

Over the past few decades, industrial land policies in China have undergone substantial changes, responding to both domestic and global pressures such as limited land resources, green transformation and upgrading, and the reshoring of manufacturing internationally. These shifts have been especially pronounced in Zhejiang Province, where local government competition, climate commitments, and the rise of small and micro-enterprises have collectively reshaped the industrial development.

### Early phase: local government competition and low-density industrial land use

In the early stages of China's industrialization, particularly from the 1980s to the early 2000s, local governments engaged in fierce competition to attract investment by offering industrial land at low prices. Rapid economic growth was prioritized, with land leveraged as a key incentive to draw businesses in China's economic reforms. Studies have demonstrated how this "race to the bottom" in land pricing led to industrial land inefficiencies, with industrial land prices set far below market levels [39].

In regions like Zhejiang, as elsewhere in China, the emphasis of industrial land policy was placed on the quantity of industrial land allocation rather than its efficiency, mirroring trends in other developing economies where governments prioritize industrial expansion over sustainable land management [40]. Such practices also caused extensive overuse of land, as exemplified in China's Pearl River Delta, where low land prices served as a magnet for foreign direct investment but generated high social and environmental costs [41]. By the early 2000s, however, concerns about the inefficiencies and environmental consequences of these policies began to emerge, prompting calls for reform to address the long-term sustainability of industrial land use [36].

### Policy shifts in response to global climate commitments

Since the late 2000s, China has progressively reformed its industrial land-use policies in response to mounting environmental concerns and global climate commitments. The Paris Agreement established global objectives to limit temperature rise, with China committing to peak carbon emissions by 2030 and achieving carbon neutrality by midcentury [42]. By 2030, national spatial planning aims to limit urban growth to 116,700 km^2^, permitting the development of just 27,700 km^2^ of new urban construction land between 2016 and 2030 [43]. In line with these targets, Zhejiang Province introduced the "Standard Land" (Biaozhun Di) when transferring industrial land policy in 2016, which soon expanded nationally. This policy standardized the land leasing process, incorporating stricter requirements for FAR, energy efficiency, and environmental standards, thereby enhancing transparency and fairness in industrial land allocation

### Recent developments: growth of small and micro-enterprises (SME) parks and M0 industrial land use

**Fig 2:**
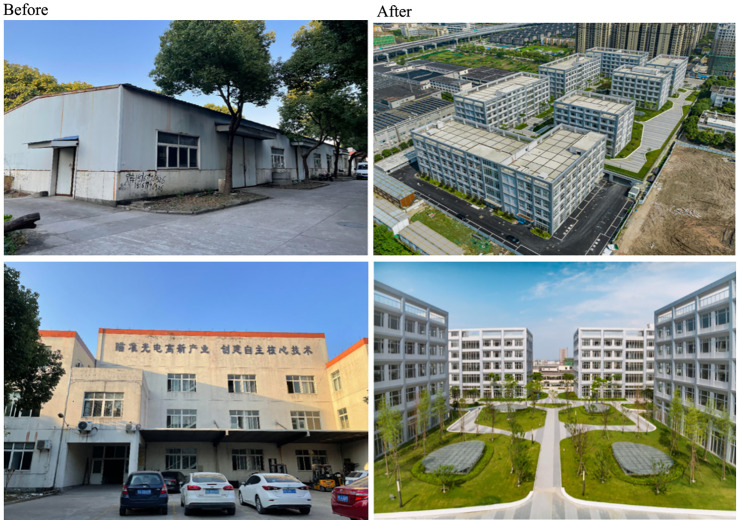
Redevelopment of Linping Jin Cheng Technology Industrial Park, Hangzhou City, Zhejiang (FAR increased from 1.0 to 2.66. Source: Linping Jin Cheng Technology Industrial Park).

In recent years, the rapid growth of SMEs and rising land prices have driven the development of SME industrial parks to accommodate these businesses. Zhejiang has taken the lead in addressing the issue of "low, small, scattered" enterprises by implementing a policy to phase out underperforming businesses with annual tax revenues below 10,000 RMB per mu (approximately $2/m²). The province plans to establish 1,200 new SME industrial parks to transition from fragmented, inefficient land use to more concentrated, high-density industrial parks. Unlike earlier phases of industrial development, the newly developed parks are operated by professional firms that acquire land and manage industrial properties, providing well-serviced spaces that small and micro enterprises can either purchase or lease. For example, the Jin Cheng International Science and Technology Industrial Park, located in the Linping Economic and Technological Development Zone in Hangzhou, spans 94 acres. After renovation and reconstruction in 2019, the floor area ratio was increased from 1.0 to 2.66. The park focuses on biopharmaceuticals while also accommodating light industry and technology-based enterprises (Fig 2).

Furthermore, as China shifted towards an innovation-driven economy, traditional industrial land classifications became inadequate for high-tech and creative industries. To address this, the "Innovative Industrial Land (M0)" category was introduced. This new classification integrates manufacturing with research and development, supporting mixed-use developments to foster innovation hubs. As part of national strategies like Made in China 2025, this initiative aims to promote advanced manufacturing and drive technological innovation.

## Methodology

### Description of spatiotemporal characteristics

Two methods have been employed to depict the spatiotemporal characteristics of the new industrial land FAR. The first method employs ArcGIS's trend analysis tool to generate a three-dimensional global trend map showing the distribution of industrial land FAR across counties (cities, districts) from 2019 to 2023 in Zhejiang, China. In this map, the X-axis corresponds to the east direction, with the green line depicting the trend in the east-west direction. The Y-axis represents the north direction, with the blue line illustrating the trend in the north-south direction. The Z-axis indicates the FAR values. The second method used is the standard deviation ellipse, which helps to analyze the spatial distribution and concentration of industrial land FAR across the study area. The standard deviation ellipse provides a statistical representation of the spatial dispersion and central tendency of the FAR data, showing the orientation, shape, and extent of the industrial land concentration. It allows for the identification of areas with high and low FAR values and helps in understanding the spatial organization and concentration patterns of industrial land use.

### Construction of the model and identification of the variable

Building on the theoretical analysis above, this study employs a panel data model to examine the factors influencing the FAR of industrial land. We employ an OLS model with year fixed effects. OLS has been widely adopted in the literature on industrial land prices and land-use intensity [3], and standard errors are clustered at the county level to account for within-county correlation. The linear panel data regression is specified as follows:


$${LnFAR}_{it}=c+\alpha {LnX}_{it}+\beta {lnZ}_{it}+\lambda_{t}+\varepsilon_{it}$$

The formula represents the basic model used to examine the effects of the factors influencing the FAR of new industrial land. The subscripts i and t  represent the county and the year, respectively. $\lambda_{t}$ captures the year fixed effects, and $\varepsilon_{it}$ is the random error term. *α* and *β* are the parameters to be estimated. The variance decomposition indicates that between-county variance accounts for 81.98% of the total variation in LS, while within-county variance accounts for only 18.02%. This suggests that LS varies mainly across counties rather than over time. Including county fixed effects would therefore absorb most of its explanatory power. Accordingly, the baseline specification incorporates only year fixed effects.

(1) Dependent variable: FAR represents the spatial density of new industrial land from 2019 to 2023. In China, urban construction land is state-owned, and industrial enterprises acquire land use rights from the government for up to 50 years by paying land transfer fees. The government sets conditions, including minimum price, FAR, and industry type, in an online land market where enterprises compete for land. Enterprises can build industrial buildings with a FAR higher than the minimum, and the government does not require additional fees for the increased building area to encourage land intensification. The logarithmic transformation of FAR is employed to reduce skewness, as the variable exhibits an approximately normal distribution after transformation (Table 1).
(2) Core independent variables: X_it_ represents the core independent variables, including the total industrial land supply area of each year (LS), the land transfer price (LP) and per capita arable land area (AL), which represent the impact of the government land supply quota, the industrial land transfer price, and land resource conditions. While the government sets a minimum price for land transfer, industrial land must be publicly competed for, meaning the actual price may exceed the minimum. This is particularly a new phenomenon in Zhejiang in recent years, where high land transfer transparency and strong demand from small and micro-sized enterprises exist (Table 1).
(3) Control independent variables: Z_it_ represents the control variables. Per capita GDP (GDP) reflects the economic development level of each region. Transportation accessibility is captured through two measures: the distance to the nearest major transport hub (airport, railway station, or toll station, D_Station) and the count of highways and arterial roads within a 3 km radius (N_Highway), both of which significantly influence land use and economic activity [44,45]. Use the distance to government centers (D_Center) as a proxy for the relative location within the city [45]. Industrial agglomeration is measured by the number of industrial enterprises within 3 km (N_firm), reflecting clustering effects. Plot factors refer to the individual plot area transferred (PA) and investment intensity (Invest). The latter represents the government's requirements when transferring industrial land, which vary by industry type. It specifies the minimum fixed asset investment per unit of land area, including the costs of land purchase, factory construction, and machinery and equipment purchases (Table 1).

**Table 1:** List of variable definitions.

	Variable Name	Variable Definition	Variable Description	Expected Sign
Core independent variables	Land Price	LP	land price of individual new industrial land (in 10,000 CNY/m²)	+
	Land Supply Area	LS	Total industrial land transfer area in each county or district per year（in 10000m²）	±
	Per Capita Arable Land Area	AL	Per capita arable land area (in mu per person, where 1mu ≈ 666.67 m²) for each county or district	–
Control independent variables	Per Capita GDP	GDP	Per capita GDP (CNY) of the county or district where the land parcel is located in the year of transfer	+
	Distance to City Center	D_Center	Distance from the plot to the local government (m)	–
	Distance to Transport Nodes	D_Station	Distance to the nearest airport, railway station, and toll station entrance (m)	–
	Number of Nearby Highways	N_Highway	Count of urban arterial roads and highways within a 3 km surrounding the industrial land plot	+
	Industrial agglomeration	N_Firm	Count of industrial enterprises within a 3 km surrounding the industrial land plot	+
	Plot Area	PA	Area of the new industrial land plot（m²）	–
	Investment Intensity	Invest	Fixed asset investment per mu (10,000 CNY/mu)	+

To enhance the stability and interpretability of the model, both the core explanatory variables and control variables included in the analysis are transformed using natural logarithms. Table 2 reports the descriptive statistics for all variables.

**Table 2:** Descriptive statistics.

Variables	Observations	Mean	Standard Deviation	Minimum	Maximum
FAR	10601	1.3	0.4	0.01	6.9
LS	10601	140.8	121.9	0.28	585.1
LP	10601	1055	3997	4.7	167821
Farmland	10601	0.4	0.2	0	2.1
GDP	10601	105762	39928	38865	486954
D_Center	10601	11609	6538	779	77160
D_Station	10601	4655	3651	147	79190
N_Highway	10601	228.7	794.8	0	25202
N_Firm	10601	6.5	8.3	0	100
PA	10601	27320	43324	19	1000000
Invest	10601	375.1	244.9	22	10000

Furthermore, recognizing the sectoral heterogeneity, this study also incorporates industry-specific factors. Significant variations exist across different industrial sectors in terms of production processes, equipment requirements, and environmental standards, all of which directly impact land use intensity and spatial layout demands. In this study, the industry classification is based on the *Industrial Classification for National Economic Activities* (2017), supplemented by the *Classification of High-Technology Industries (Manufacturing)* (2017) and the *Zhejiang Province High-Tech Industry (Manufacturing) Statistical Classification Directory* (2018) (S2 Table).

Multicollinearity among the independent variables was assessed using the Variance Inflation Factor (VIF). The diagnostic results revealed that all variables had a VIF value below 5, indicating that multicollinearity was not a concern. To test for heteroscedasticity, the White test was conducted. A p-value lower than 0.05 led to the rejection of the null hypothesis of homoscedasticity, suggesting the presence of heteroscedasticity in the data. As a result, robust standard errors were employed in the regression analysis to improve the accuracy and reliability of the parameter estimates, thereby mitigating potential biases associated with heteroscedasticity.

### Case context

**Fig 3:**
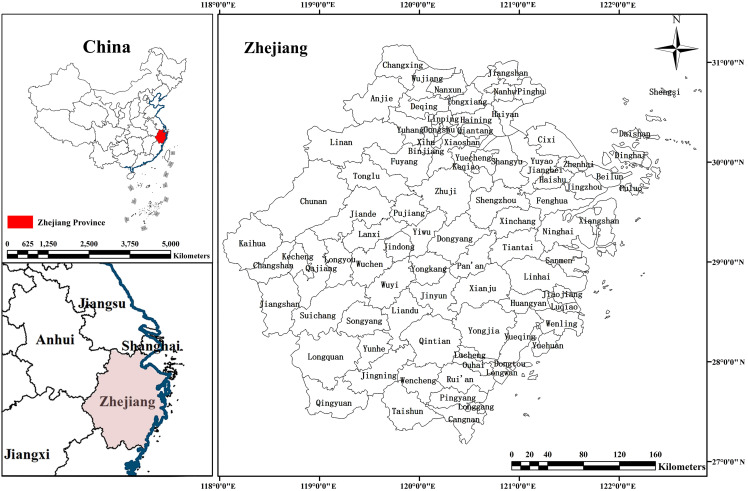
Study area.

Zhejiang Province is situated in the south of the Yangtze River Delta, its northern borders connect with Shanghai and Jiangsu Province (Fig 3). The provincial territory, spanning 105,500 km^2^, exhibits distinct geomorphological characteristics, with mountainous topography covering 74.6% of the terrain, aquatic systems accounting for 5.1%, and plains making up the remaining 20.3%. Zhejiang ranks fourth in industrial-added value among all provincial-level administrative regions in China. In 2023, Zhejiang's Gross Domestic Product (GDP) reached 8.3 trillion yuan (approximately 1.16 trillion USD), with a three-sector industrial structure of 2.8:41.1:56.0. The province is particularly noted for its robust base of small and micro-enterprises, which have played a crucial role in driving innovation and economic growth. Zhejiang is home to 56,828 industrial enterprises above a designated size with annual primary business revenues exceeding 20 million RMB (approximately 2.8 million USD), with 84% of these being private enterprises. Additionally, Zhejiang is one of China's leading marine economy provinces, with Ningbo-Zhoushan Port holding the top spot in global cargo throughput for 15 consecutive years and ranking third in global container throughput.

### Data sources and data processing

The data on newly added industrial land comes from the website of Zhejiang Provincial Department of Natural Resources. Annual GDP and permanent population data for counties (cities and districts) in Zhejiang Province were derived from Zhejiang's statistical yearbooks and government websites. Geographic data for counties (districts) were obtained from Google Earth, while information on roads, airports, railway stations, toll stations, and industrial enterprises was collected from AMap using POI-capture software. The dataset incorporates both coordinate-based location information and transportation-network distances, sourced through AMap's open online services. All data collection and processing strictly adhered to AMap's terms of use, and no personal or sensitive information was accessed or collected. The analysis was conducted in full compliance with the platform's data usage policies. After eliminating missing data, the neighbor analysis and buffer spatial link tools in ArcGIS were used to calculate the distance between the plot and the city center, the distance to the transportation hub, the number of surrounding roads, and the number of surrounding industrial enterprises. Samples lacking geographical location information or key characteristic variables were excluded. After this filtering, 10,601 valid samples were retained, representing 90.4% of the total sample.

## Results and analysis

### The spatiotemporal characteristics

**Fig 4:**
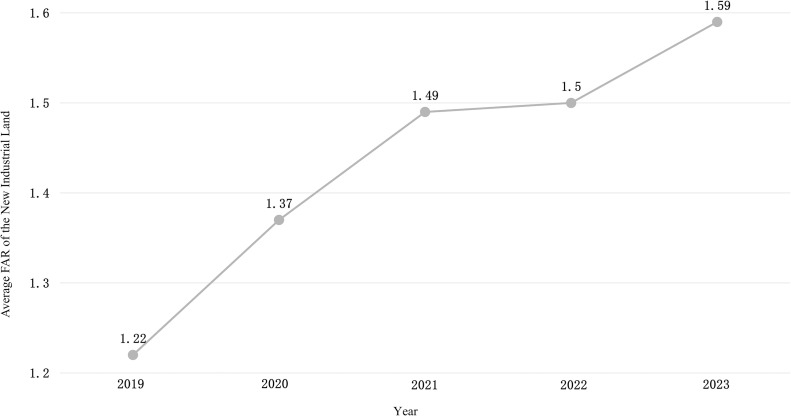
Average FAR for new industrial land in Zhejiang from 2019 to 2023.

**Fig 5:**
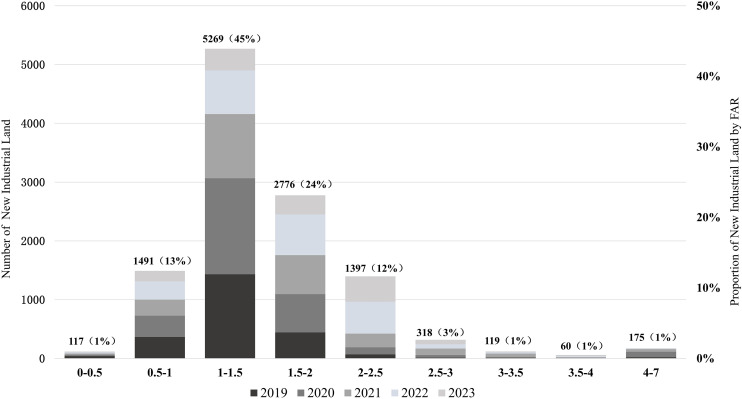
Distribution of FAR for new industrial land in Zhejiang Province from 2019 to 2023.

From a temporal perspective, the FAR of new industrial land in Zhejiang Province exhibited an upward trend. From 2019 to 2023, the province's average industrial land FAR increased from 1.22 to 1.59, with an average annual growth rate of 6.8% (Fig 4). Despite a slowdown in growth from 2021 to 2022, the overall trend remained steadily upward, highlighting a substantial increase in the intensive utilization of industrial land. From 2019 to 2023, FARs were predominantly concentrated in the 1.0–1.5 range, accounting for 45% of all plots. This was followed by the 1.5–2.0 range, representing 24% of the plots. A smaller number of plots exhibited higher FARs, with 354 plots (3%) exceeding 3.0 and 175 plots (1%) exceeding 4.0 (Fig 5).

**Fig 6:**
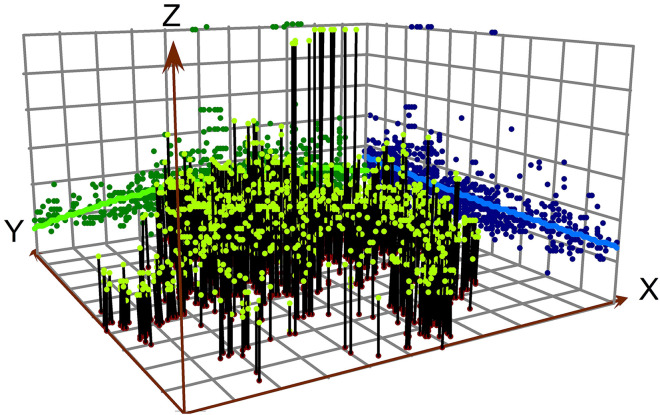
Trend surface of FAR for new industrial land in 2019.

**Fig 7:**
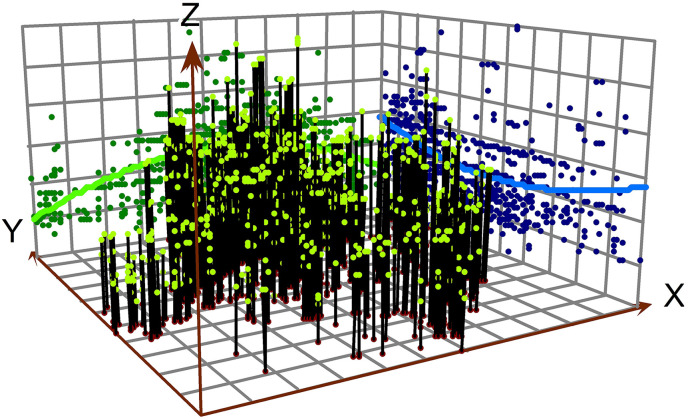
Trend surface of FAR for new industrial land in 2023.

**Fig 8:**
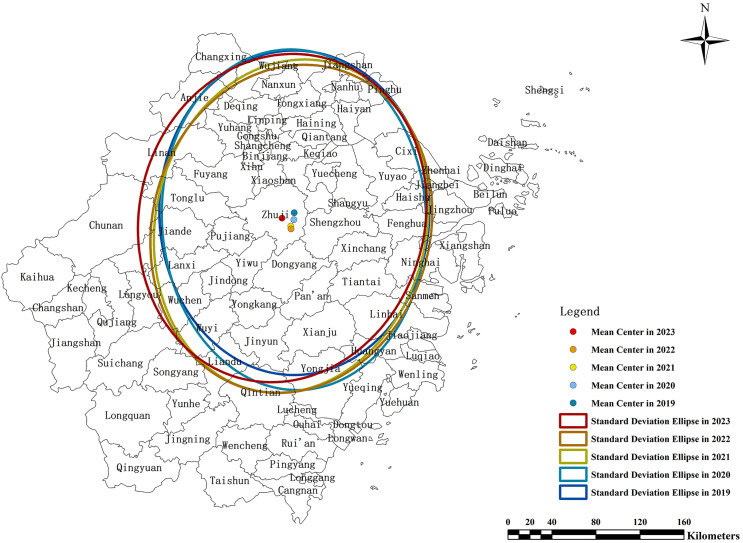
Standard deviation ellipse of FAR for new industrial land from 2019 to 2023.

The spatial distribution of FAR for industrial land in Zhejiang Province shows the highest levels in the northern coastal cities Hangzhou and Ningbo, a mid-range peak around Jinhua, and relatively lower but rising values toward the southern areas including Wenzhou. The trend surface maps for 2019 and 2023 show that high FAR values are concentrated in central Zhejiang, particularly around Jinhua and its neighboring counties. Between 2019 and 2023, the most pronounced increases occurred in northern and southern areas, indicating a pattern of spatial convergence in industrial development intensity (Fig 6 and 7). This pattern reflects the economic influence of central Zhejiang, driven by industrial hubs like Yiwu—home to the world's largest offline small commodity market—has played a significant role in stimulating surrounding industrial development. Additionally, the notable growth in the northern and southern regions can be attributed to the "Hangzhou-Ningbo-Shaoxing-Jiaxing" metropolitan cluster, anchored by Hangzhou as the provincial capital, and to Wenzhou's status as the most densely concentrated region for private enterprises in China. Standard deviational ellipse analysis further indicates that the centroid of industrial land FAR shifted southward. By 2023, the centroid moved slightly northward, suggesting a renewed increase in industrial land FAR in northern Zhejiang (Fig 8).

### The industrial land FAR for various subsectors and regions

**Fig 9:**
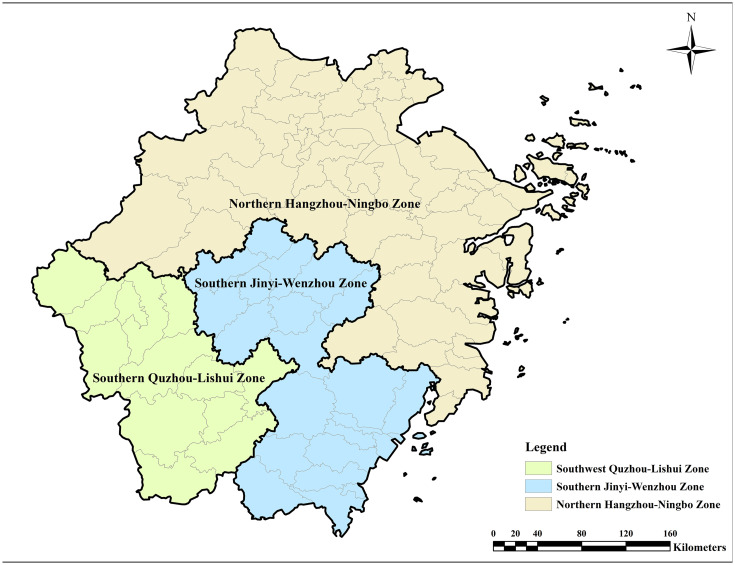
Regional division of Zhejiang Province.

Based on the Zhejiang Provincial Territorial Spatial Plan (2021–2035), Zhejiang is divided into three major regions: (1) the Northern Hangzhou-Ningbo Zone, covering seven prefecture-level cities-Hangzhou, Ningbo, Jiaxing, Huzhou, Shaoxing, Zhoushan, and Taizhou; (2) the Southern Jinhua-Wenzhou Zone, including Jinhua and Wenzhou, and parts of Lishui; and (3) the Southwestern Quzhou-Lishui Zone, consisting Quzhou and Lishui (Fig 9).

**Fig 10:**
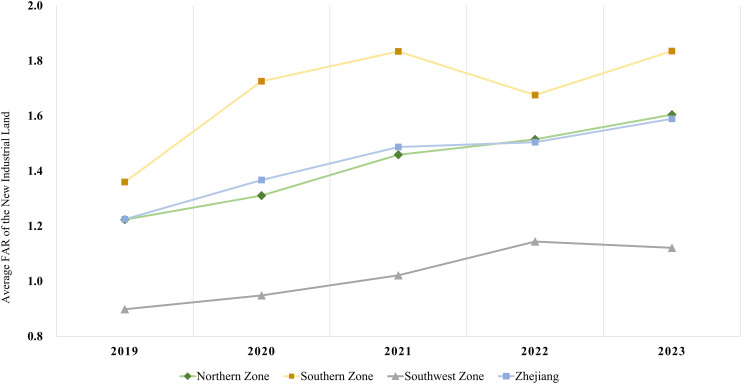
The regional average FAR of new industrial land in Zhejiang from 2019 to 2023.

From 2019 to 2023, average FARs increased from 1.22 to 1.60, 1.36 to 1.84, and 0.90 to 1.12, respectively. The industrial land FAR gap between regions initially widened and then narrowed. The southwestern zone's FAR remained below the provincial average, the northern zone stayed near the average, and the southern area was well above it (Fig 10), following the pattern: Southern > Northern > Southwestern. The southern region recorded the highest average annual FAR growth at 7.8%, while the northern region followed with a relatively lower rate of 5.7%.

**Fig 11:**
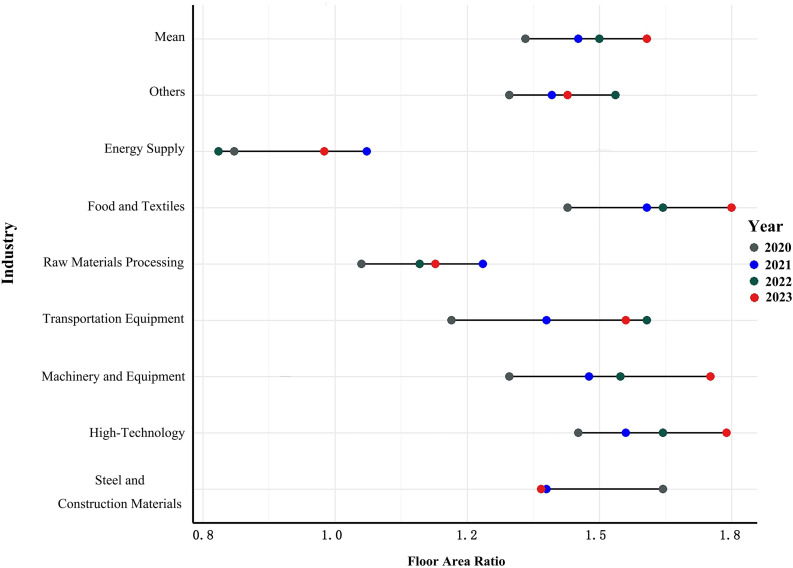
Annual variation in FAR for new industrial land by industry from 2020 to 2023.

Due to missing industry classification data for 2019, this study analyzes the FAR characteristics and changes of industrial land by sector from 2020 to 2023. The high-tech, food, and textile industries had the highest average FAR values, at 1.59 and 1.57, respectively, surpassing the overall industry average of 1.46. Sectors like energy, raw material processing, and steel and building materials showed more fluctuation in FAR, with a declining trend. Other industries generally experienced an upward trend in FAR (Fig 11).

### The influence factors of industrial land FAR

The regression results are shown in Table 2. For the full sample in Model 1, Land Price (LP) (For the sake of readability, all log-transformed variables (e.g., ln_LP, ln_LS, and similar) are referred to in abbreviated form as LP, LS, etc., throughout the text.) is not statistically significant. Land Supply (LS) has a significant positive impact on FAR, indicating that industrial land is still in a state of scarcity. The Per Capita Arable Land Area (AL) shows a negative correlation, with a coefficient of −0.151, suggesting that the natural endowment of land resources significantly affects FAR. In areas with less arable land and higher proportions of mountainous terrain, the FAR tends to increase (Table 3).

**Table 3:** Regression results of new industrial land FAR in Zhejiang Province and subregions.

Variables		Zhejiang	Regions		
			Northern Zone	Southern Zone	Southwest Zone
		Model 1	Model 2	Model 3	Model 4
Core independent variables	Ln_LP	0.008	0.002	0.010*	−0.008
		(1.547)	(0.349)	(1.839)	(−0.575)
	Ln_LS	0.009***	0.011***	−0.004	−0.018
		(2.656)	(3.240)	(−0.273)	(−1.077)
	Ln_AL	−0.151***	−0.127	−0.005	−0.137
		(−2.694)	(−1.612)	(−0.064)	(−0.951)
Control independent variables	Ln_GDP	−0.001	−0.038	0.177***	0.007
		(−0.024)	(−1.097)	(2.820)	(0.078)
	Ln_D_Center	−0.004	−0.023***	−0.011	0.063***
		(−0.408)	(−2.678)	(−0.699)	(3.291)
	Ln_D_Station	−0.011**	−0.013**	0.006	−0.021
		(−2.183)	(−2.104)	(0.709)	(−1.749)
	Ln_N_Highway	0.008**	0.007*	0.009	0.007
		(2.373)	(1.736)	(1.424)	(0.812)
	Ln_N_Firm	0.012***	0.012***	0.008	0.027**
		(3.201)	(3.057)	(0.895)	(2.347)
	Ln_PA	−0.006	−0.006	−0.003	0.009
		(−1.282)	(−0.949)	(−0.571)	(1.367)
	Ln_Invest	0.114***	0.115***	0.072***	−0.020
		0.008	0.002	0.010*	−0.008
	Year	Yes	Yes	Yes	Yes
	Constant	0.213***	0.913*	−1.568**	0.353
		(2.652)	(1.918)	(−2.156)	(0.348)
N		10601	7342	2217	1042
R2		0.237	0.228	0.223	0.105
Adj. R2		0.236	0.226	0.218	0.092
F-statistic		23.39	13.38	24.43	5.24

Note: ***, **, * denote significance at the 1%, 5%, and 10% confidence levels.

Among the control variables, highway accessibility (N_Highway) positively affects FAR, indicating that better transport connections enhance land intensity. Proximity to stations (D_Station) suggests that sites nearer to transport hubs tend to have higher FAR. Industrial agglomeration (N_Firm) raises density, with a 1% increase in nearby firms linked to a 0.012% rise in FAR. At the plot level, investment intensity (Invest) shows a strong positive effect, implying that greater investment per unit of land fosters more intensive and vertical development.

For the regional samples, the regression results revealed significant differences in the influencing factors across the three regions. In the southern region, where land prices are highest, industrial land prices (LP) have a significant positive impact on FAR. However, in the northern region with moderate prices and the southwestern region with the lowest prices, this effect is not significant. Industrial land supply (LS) shows a significant positive correlation in the northern region, but in the less developed southwestern region, its effect is negative. This strong regional heterogeneity suggests that industrial land supply is closely related to market demand and differences in industrial sector types across regions. Per Capita Arable Land Area (AL) shows a significant negative effect in the overall provincial model but becomes insignificant in subregional estimations. The loss of significance suggests that its impact may depend on regional conditions or industrial structures.

Lagged county-level averages of land price (LP) and land supply (LS) are used to address potential simultaneity. Aggregating to the county level reduces noise from parcel-level transactions and mitigates endogeneity due to idiosyncratic shocks. Table 4 shows results similar to the baseline estimates. The coefficients on lagged LP and LS are positive, with lagged LS statistically significant, indicating a persistent effect of land supply on industrial land use intensity (S1 Table). Control variables such as per capita farmland, industrial agglomeration, and investment intensity remain stable, consistent with the main findings. A spatial autocorrelation test on the model residuals, based on a county-level spatial weights matrix, yields a non-significant Moran's I statistic (p = 0.555), indicating no systematic spatial dependence in the unobserved components.

**Table 4:** Baseline and Robustness Regression Results.

	(1)	(2)
	Model 1	Robustness Model
Ln_LP	0.008	
	(1.547)	
Ln_LS	0.009***	
	(2.656)	
Ln_AL	−0.151***	−0.161**
	(−2.694)	(−2.305)
Ln_GDP	−0.001	−0.001
	(−0.024)	(−0.046)
Ln_D_Center	−0.004	−0.012
	(−0.408)	(−1.207)
Ln_D_Station	−0.011**	−0.012**
	(−2.183)	(−2.115)
Ln_N_Highway	0.008**	0.010***
	(2.373)	(2.681)
Ln_N_Firm	0.012***	0.013***
	(3.201)	(2.911)
Ln_PA	−0.006	−0.006
	(−1.282)	(−1.342)
Ln_Invest	0.114***	0.110***
	(8.751)	(7.828)
Ln_LP_lag		0.023
		(1.522)
Ln_LS_lag		0.000**
		(2.166)
Year	Yes	Yes
Constant	0.257	0.325
	(0.783)	(0.779)
N	10601	7952
R^2^	0.237	0.237
Adj. R^2^	0.236	0.236
F-statistic	23.39	19.44

Note: ***, **, * denote significance at the 1%, 5%, and 10% confidence levels.

Table 5 shows variations in influencing factors across industries. Land Price (LP) shows a significant positive impact on FAR in the machinery and equipment, steel and building materials, and high-tech industries. More sensitive to land prices, high-tech industries rely on superior locational conditions that offer talent concentration, knowledge spillovers, and high-quality infrastructure. In the machinery and equipment industry, enterprises tend to pursue compact layouts and multi-story factory designs to reduce production and logistics costs under rising land prices. The steel and building materials sector reflects similar pressure for spatial efficiency. Land Supply (LS) is significant in steel and building materials, food and textiles, and other industries. Per Capita Arable Land Area (AL) consistently shows a negative impact on FAR across industries, in line with the overall model.

**Table 5:** Regression results by industry subsectors.

	(1)	(2)	(3)	(4)	(5)	(6)	(7)	(8)
	Transportation Equipment	Raw Materials Processing	Machinery and Equipment	Energy Supply	Steel and Building Materials	Food and Textiles	High-Tech	Others
Ln_LP	0.016	0.021	0.031***	0.026	0.024**	0.002	0.035***	−0.004
	(0.387)	(0.294)	(0.006)	(0.459)	(0.011)	(0.822)	(0.004)	(0.642)
Ln_LS	0.041***	−0.022	0.014	−0.054	0.012***	0.012**	0.021**	0.019
	(0.028)	(0.148)	(0.137)	(0.332)	(0.000)	(0.019)	(0.028)	(0.165)
Ln_AL	−0.082	−0.176	−0.086	−0.125	−0.160**	−0.151**	−0.073	−0.223***
	(0.418)	(0.181)	(0.292)	(0.536)	(0.039)	(0.020)	(0.339)	(0.000)
Ln_GDP	−0.044	0.030	−0.038	−0.015	0.010	0.018	−0.003	−0.042
	(0.307)	(0.445)	(0.251)	(0.889)	(0.796)	(0.565)	(0.957)	(0.309)
Ln_D_Center	−0.009	0.022	−0.006	0.159*	−0.010	−0.009	−0.026*	−0.004
	(0.598)	(0.412)	(0.671)	(0.065)	(0.385)	(0.373)	(0.059)	(0.783)
Ln_D_Station	−0.022	−0.014	−0.007	−0.010	−0.021**	0.002	−0.014*	−0.018*
	(0.187)	(0.216)	(0.421)	(0.869)	(0.012)	(0.734)	(0.094)	(0.077)
Ln_N_Highway	−0.000	0.009	0.005	0.020	−0.003	0.013**	0.002	0.013*
	(0.994)	(0.157)	(0.341)	(0.302)	(0.472)	(0.023)	(0.784)	(0.072)
Ln_N_Firm	0.021**	0.004	0.021***	0.055	−0.006	0.011*	0.004	0.012
	(0.013)	(0.653)	(0.003)	(0.269)	(0.380)	(0.096)	(0.504)	(0.172)
Ln_PA	−0.001	−0.027***	0.000	−0.014	0.002	0.003	−0.004	−0.002
	(0.924)	(0.000)	(0.951)	(0.510)	(0.658)	(0.680)	(0.516)	(0.723)
Ln_Invest	0.130***	0.046*	0.149***	0.067	0.172***	0.078***	0.114***	0.099***
	(0.000)	(0.096)	(0.000)	(0.421)	(0.000)	(0.000)	0.035***	(0.000)
Year	Yes	Yes	Yes	Yes	Yes	Yes	Yes	Yes
Constant	0.507	0.284	0.217	−0.930	−0.219	0.128	0.270	0.860*
	(0.404)	(0.655)	(0.627)	(0.518)	(0.673)	(0.755)	(0.594)	(0.050)
Observations	528	862	1,508	69	992	1,378	1,807	717
R-squared	0.251	0.146	0.266	0.305	0.293	0.241	0.247	0.242

Note: ***, **, * denote significance at the 1%, 5%, and 10% confidence levels, respectively.

Control variables exhibit distinct effects: the distance to city centers (D_Center) has a significant positive effect on FAR in energy supply and a negative one in high-tech, showing divergent spatial preferences. The negative association for high-tech firms indicates a stronger sensitivity to proximity to central business districts, where access to innovation resources, skilled labor, and business services fosters more intensive land use. Closer to transport nodes (D_Station) significantly increase FAR in steel and building materials and high-tech sectors, confirming that accessibility promotes vertical development. The number of nearby highways (N_Highway) positively influences FAR in food and textiles and other industries, while industrial agglomeration (N_Firm) significantly increases FAR in transportation equipment, machinery and equipment, and high-tech industries, highlighting the clustering effect of manufacturing activities.

## Discussion

The analysis conducted in the previous section and the findings generated in this study provide more clarity to the functional relationship between FAR, price, and land supply. Overall, Baseline estimates show no general effect of land prices on industrial FAR; price-induced intensification is confined to high-price regions, SME-dense areas, and selected sectors such as high-technology, machinery and equipment, and steel and building materials. In contrast, natural land availability and government-regulated supply differ by economic development level—natural land supply remains consistent across regions, whereas government regulations create variation.

### Responding to regional land scarcity through vertical development strategies

**Fig 12:**
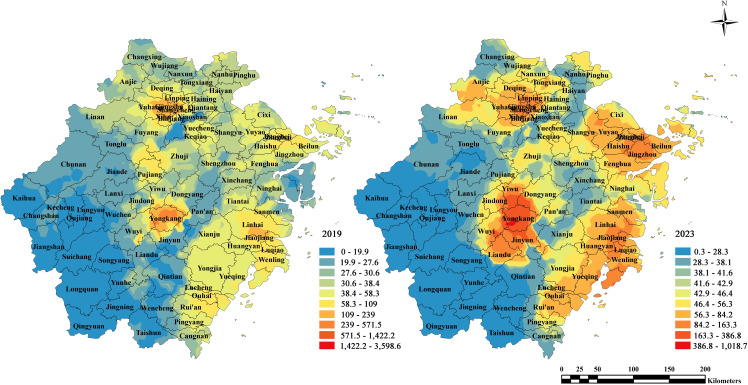
Heat map of land price of new industrial land in Zhejiang from 2019 to 2023.

Studies show that land price is a key driver of increasing FAR; however, its impact varies significantly across regions due to differences in the scale and number of enterprises. The FAR of industrial land in the southern region of Zhejiang is the most responsive to land prices. The southern area hosts a dense concentration of small and micro enterprises. Jinhua and Wenzhou, the two core cities in the south, are symbolic of China's reform and opening-up, with Jinhua leading in the global small commodity market and Wenzhou in private enterprise development. From 2019 to 2023, Zhejiang recorded 175 industrial land transactions with a minimum FAR of 4 or higher, 81.7% of which were located in Yongkang, Jinhua, where the average land price reached $364.58/m² (1.75million yuan/mu)—4.3 times the provincial average. There were also 179 transactions with a minimum FAR between 3 and 4, mainly concentrated in Yiwu (Jinhua), Xiaoshan (Hangzhou), and Ruian and Ouhai (Wenzhou). In contrast, the southwestern region of Zhejiang, which is relatively less developed, does not exhibit a positive correlation between FAR and land price. This suggests that to attract investment, local governments in less developed areas may still offer lower land prices as an incentive (Fig 12).

However, the sustained rise in industrial land prices is not conducive to industrial development, as high land costs significantly increase production expenses for enterprises, thereby squeezing out funds available for innovation and R&D investments [2,6,7]. In the long run, this situation undermines regional industrial competitiveness. Areas characterized by high land prices typically face shortages in land resources, necessitating solutions through expanding land supply and promoting vertical industrial building development. In pursuing vertical development, enhancing the adaptability and suitability of industrial buildings becomes crucial. The developers must focus on innovative design approaches, improve load-bearing capacity, optimize functional layouts, and provide flexible spatial modules to accommodate diverse industrial requirements.

### Addressing sectoral needs and spatial divergence in the manufacturing

Since 2022, Zhejiang Province has actively promoted the use of multi-story industrial buildings, primarily targeting high-tech and light manufacturing sectors. While this initiative has generally increased FAR across various industries, its effects vary significantly by sector. In high-tech industries, transportation equipment, food and textiles, and steel and building materials, the rise in FAR despite expanded land supply can be attributed to strong policy mandates for land-use intensification, the spatial needs of technology- and capital-intensive industries, and persistent agglomeration forces that motivate firms to adopt denser development forms.

These patterns indicate that increases in industrial FAR are driven by distinct sector-specific development logics rather than a uniform process of industrial intensification. High-tech industries rely heavily on the agglomeration of R&D support and producer services, while food and light manufacturing activities are generally well suited to multi-story factory buildings, where compact spatial layouts support standardized production and service integration. Regression results further indicate that not all industrial sectors are suited to vertical development strategies. Raw material processing industries, in particular, exhibit limited potential for FAR increases due to the spatial rigidity imposed by production processes and the need to accommodate bulk operations and operational safety requirements. Therefore, while technology-intensive sectors adapt well to compact, multi-story layouts, several manufacturing activities continue to require extensive horizontal space to ensure production efficiency and risk mitigation.

These findings suggest that the implementation of “industrial-upstairs” policies should be carefully calibrated to sectoral characteristics and spatial requirements, rather than applied as a one-size-fits-all approach. Vertical industrial development should be prioritized in R&D-oriented activities and light manufacturing sectors, accompanied by refined technical and spatial guidelines tailored to specific industries, including building structure design, load-bearing standards, and environmental protection requirements.

### Promoting market-oriented industrial land allocation and the efficiency implications of policy-driven FAR controls

When terrain factors significantly positively influence FAR, government regulations generate a counterintuitive outcome, whereby increases in annual industrial land supply are associated with higher FAR values. This finding contradicts traditional market logic, which suggests that increased land supply reduces scarcity and lowers FAR values, differing from the inverse correlation commonly observed in residential land use studies [46] The delayed responsiveness of FAR adjustments to land supply variations indicates that the industrial land market exhibit a mismatch between supply and demand. In China，the vast majority of industrial land has only one company participating in the bidding, while in the residential land market, except during periods of sluggish market demand, land competition is generally strong at other times. The low competitiveness of industrial land is not due to a lack of demand from enterprises, but rather because the government imposes additional conditions when transferring industrial land, such as industry type, company size, and so on. Therefore, enhancing market-driven allocation processes is essential for establishing a more timely and rational relationship between changes in land supply and FAR adjustments.

The increase in industrial FAR observed in this study reflects the joint influence of market forces and administrative mandates, although their relative importance varies across regions and sectors. In areas where industrial FAR exhibits high sensitivity to land prices, FAR adjustments are largely market-driven responses to rising land scarcity and land costs. In such contexts, firms voluntarily adopt more intensive development patterns in order to economize on land inputs and maintain production efficiency. By contrast, in regions where FAR continues to rise despite expanded industrial land supply, the results suggest a stronger role of administrative planning instruments. Mandatory FAR requirements embedded in land transfer conditions and industrial land-use regulations can induce vertical development even when market pressures are relatively weak. This pattern deviates from conventional market logic, in which increased land supply would be expected to alleviate scarcity and moderate development intensity.

This divergence has important policy implications. When policy-driven FAR targets exceed actual market demand or the operational requirements of specific industries, firms may pursue higher-density development primarily to satisfy regulatory constraints rather than to optimize production efficiency. Such misalignment may lead to underutilized built space, higher construction and operating costs, and reduced flexibility in industrial operations. These findings underscore the need for a more balanced approach to industrial intensification, in which administrative FAR controls are better aligned with market conditions and sector-specific production characteristics.

From a broader perspective, the tension between land scarcity and industrial densification observed in Zhejiang Province reflects a structural challenge commonly faced by emerging economies and high-density urban regions. First, promoting land-use intensification through the development of SME industrial parks and vertical development can simultaneously improve land-use efficiency and better accommodate the spatial needs of small and micro enterprises, which often exhibit high sensitivity to land prices and limited access to land resources. Second, the results highlight the importance of adopting industry-specific land-use access criteria and intensity requirements, rather than uniform FAR targets, to reflect heterogeneity in production processes, spatial needs, and operational risks across industrial sectors. Third, enhancing the fairness and competitiveness of industrial land transfers—by reducing excessive administrative restrictions and encouraging broader market participation—can help align land allocation outcomes more closely with actual industrial demand.

## Conclusion

This research used the FAR as the urban industrial spatial density indices to measure and analyze the spatiotemporal characteristics, regional and industrial sub-sector's differences and influencing factors based on industrial land plots data in Zhejiang province from 2019 to 2023. Our analyses showed that the average FAR value increased from 1.23 in 2019 to 1.59 in 2023 with an average annual growth rate of 6.8%. The FAR were predominantly concentrated in the 1.0–1.5 range, accounting for 45% of all plots. The value showed a U-shape in the longitudinal direction, being higher in central Zhejiang and lower in the southern and northern regions. And it continued to increase from north to south in the latitudinal direction. The results indicate that the pattern of Southern > Northern > Southwestern with increasingly regional differences. The high-tech, food, and textile industries had the highest average FAR, while sectors like energy, raw materials, and steel/building materials exhibited greater variability and a declining trend. Most other industries experienced an increase in FAR.

The study shows that the FAR of industrial land is influenced by land prices, government-controlled land supply, and per capita arable land resources. Land prices and land supply have a positive effect on FAR, while arable land area negatively impacts it. These relationships vary by region. In the southern region, with the highest land prices, industrial land prices significantly increase FAR. Industry-specific analysis shows that land prices generally increase with FAR, with the strongest effect observed in the high-tech sectors.

The findings provide an empirically grounded basis for industrial land policies to become more flexible, market-responsive, and sector-sensitive, offering insights for countries balancing industrial growth and land scarcity under rapid urbanization. First, vertical industrial development should be prioritized in innovative industries and light manufacturing sectors, incorporating appropriate building height ratios and rigorous standards related to structural safety, load-bearing capacity, and environmental performance to ensure long-term adaptability. Second, in regions experiencing acute land scarcity and rising land costs, differentiated policy instruments are needed to support small and micro-sized enterprises, mitigating excessive cost pressures while maintaining access to industrial space. Finally, improving the efficiency of industrial land allocation requires shifting away from overly restrictive, condition-based transfer practices toward mechanisms that allow price signals and enterprise demand to play a more decisive role, thereby fostering a closer alignment between land supply, industrial needs, and actual development intensity.

The study also has several key limitations that future research could address. Future studies could incorporate polycentric urban development by analyzing FAR variations at the city or metropolitan scale, capturing how multi-nodal structures, emerging sub-centers, and intra-metropolitan spatial competition influence industrial land-use spatial density. Building on sustainability transition research, future work could also employ quasi-experimental methods to explore how industrial greening, digital transformation, and related policy interventions reshape firms' spatial organization and spatial efficiency. Such extensions would deepen understanding of how technological upgrading and policy-driven sustainability agendas interact with industrial land-use patterns and the evolving spatial structure of manufacturing.

## Supporting information

S1 Table Comparison between OLS and Fixed Effects Models. (DOCX)

S2 Table Classification of Industrial Sectors. (DOCX)

S1 Data Data and code. (ZIP)
