# Supplementary material for: Shaping industrial spatial density: How floor area ratio varies across regions and sectors in Zhejiang, China
Source: PLoS One. 2026 Mar 4;21(3):e0343089. doi: 10.1371/journal.pone.0343089 (PMC12959702; doi:10.1371/journal.pone.0343089)
Supplement: S1 Table — (DOCX) [file pone.0343089.s001.docx]

S1 Table. Comparison between OLS and Fixed Effects Models

|  | (1) | (2) |
| --- | --- | --- |
|  | OLS | FE |
| Ln_LP | 0.008*** | 0.008 |
|  | (4.160) | (1.547) |
| Ln_LS | 0.009*** | 0.009*** |
|  | (7.252) | (2.656) |
| Ln_AL | -0.151*** | -0.151*** |
|  | (-11.905) | (-2.694) |
| Ln_GDP | -0.001 | -0.001 |
|  | (-0.092) | (-0.024) |
| Ln_D_Center | -0.004 | -0.004 |
|  | (-1.207) | (-0.408) |
| Ln_D_Station | -0.011*** | -0.011** |
|  | (-5.360) | (-2.183) |
| Ln_N_Highway | 0.008*** | 0.008** |
|  | (5.056) | (2.373) |
| Ln_N_Firm | 0.012*** | 0.012*** |
|  | (6.298) | (3.201) |
| Ln_PA | -0.006*** | -0.006 |
|  | (-4.251) | (-1.282) |
| Ln_Invest | 0.114*** | 0.114*** |
|  |  |  |
| Year | YES | YES |
| Constant | 0.213*** | 0.257 |
|  | (2.652) | (0.783) |
| N | 10601 | 10601 |
| R2 | 0.237 | 0.237 |
| Adj. R2 | 0.236 | 0.236 |

Note: ***, **, * denote significance at the 1%, 5%, and 10% confidence levels, respectively.
