# Supplementary material for: Shaping industrial spatial density: How floor area ratio varies across regions and sectors in Zhejiang, China
Source: PLoS One. 2026 Mar 4;21(3):e0343089. doi: 10.1371/journal.pone.0343089 (PMC12959702; doi:10.1371/journal.pone.0343089)
Supplement: S2 Table — (DOCX) [file pone.0343089.s002.docx]

S2 Table. Classification of Industrial Sectors.

| Industry Categories | Subcategories |
| --- | --- |
| Food and Light Textile Industry | Food Manufacturing  Agro-food Processing  Textile, Apparel, and Accessories Manufacturing  Chemical Fiber Manufacturing  Furniture Manufacturing  Cultural, Educational, Sports, and Entertainment Goods Manufacturing  Printing and Recorded Media Reproduction |
| High-Technology Industry | Pharmaceutical Manufacturing  Aerospace and Aviation Equipment Manufacturing  Electronics and Communication Equipment Manufacturing  Computer and Office Equipment Manufacturing  Medical Devices and Instruments Manufacturing |
| Machinery and Equipment Industry | Specialized Equipment Manufacturing  General Equipment Manufacturing  Electrical Equipment and Machinery Manufacturing  Machinery Parts Processing  Marine Engineering Equipment Manufacturing |
| Transportation Equipment Industry | Automobile Manufacturing  Automotive Parts and Components Manufacturing  Railway, Shipbuilding, Aerospace, and Other Transportation Equipment Manufacturing  Motorcycle Manufacturing |
| Steel and Building Materials Industry | Metal Products Manufacturing  Ferrous Metal Smelting and Rolling Processing  Non-ferrous Metal Smelting and Rolling Processing  Non-metallic Mineral Products Manufacturing  Construction and Building Materials Industry  Brick, Tile, and Stone Materials Manufacturing |
| Energy Industry | Petroleum, Coal, and Fuel Processing  Refined Petroleum Products Manufacturing  Crude Oil and Petroleum Products Manufacturing  Mining and Extraction Industry  Electricity, Heat, Gas, and Water Production and Supply  New Energy and Advanced Materials Industry |
| Raw Material Processing Industry | Chemical Raw Materials and Products Manufacturing  Wood Processing and the Manufacture of Wooden, Bamboo, Rattan, Palm, and Straw Products  Rubber and Plastic Products Manufacturing  Glass Products Manufacturing  Paper and Paper Products Manufacturing |
| Other Industries | Miscellaneous Manufacturing  Comprehensive Waste Resource Utilization  Sewage Treatment and Recycling |
